# Installation of an Indole on the BRCA1 Disordered Domain Using Triazine Chemistry

**DOI:** 10.3390/biom14121625

**Published:** 2024-12-18

**Authors:** Liam E. Claton, Chrissy Baker, Hayes Martin, Sergei V. Dzyuba, Khadiza Zaman, Laszlo Prokai, Mikaela D. Stewart, Eric E. Simanek

**Affiliations:** 1 Department of Chemistry & Biochemistry, Texas Christian University, Fort Worth, TX 76129, USA; liam.e.claton@tcu.edu (L.E.C.); s.dzyuba@tcu.edu (S.V.D.); 2 Department of Biology, Texas Christian University, Fort Worth, TX 76129, USA; chrissy.baker@tcu.edu (C.B.); h.t.martin@tcu.edu (H.M.); mikaela.stewart@tcu.edu (M.D.S.); 3 Department of Pharmacology and Neuroscience, University of North Texas Health Science Center, Fort Worth, TX 76107, USA; khadiza.zaman@unthsc.edu (K.Z.); laszlo.prokai@unthsc.edu (L.P.)

**Keywords:** protein labeling, BRCA1, indole, fluorescent labels, protein modification, triazine

## Abstract

The functionalization of protein sidechains with highly water-soluble chlorotriazines (or derivatives thereof) using nucleophilic aromatic substitution reactions has been commonly employed to install various functional groups, including poly(ethylene glycol) tags or fluorogenic labels. Here, a poorly soluble dichlorotriazine with an appended indole is shown to react with a construct containing the disordered domain of BRCA1. Subsequently, this construct can undergo proteolytic cleavage to remove the SUMO-tag: the *N*-terminal poly(His) tag is still effective for purification. Steady-state fluorescence, circular dichroism spectroscopy, and isothermal titration calorimetry with the binding partner of BRCA1, PALB2, are used to characterize the indole-labeled BRCA1. Neither the reaction conditions nor the indole-tag appreciably alter the structure of the BRCA1. Mass spectrometry confirms that the target is modified once, although the location of modification cannot be determined by tandem mass spectrometry with collision-induced dissociation due to disadvantageous fragmentation patterns.

## 1. Introduction

Reactions between chlorotriazines and biomolecules have a history that spans almost seven decades. Imperial Chemical Industries (ICI) introduced reactive dichlorotriazine dyes, marketed as Procion dyes, in 1956 to dye fabrics [1,2]. Subsequently, chlorotriazines were employed in the PEGylation of proteins with the aim of understanding immunogenicity and the impact that PEG had on structure vis-à-vis function or solvent scope [3,4,5]. The PEGylation reagent was prepared by reacting trichlorotriazine with hydroxy-terminated PEG, thus leading to a dichlorotriazine or a monochlorotriazine bearing either one or two PEG chains, respectively, with the former being more reactive than the latter by virtue of the additional electronegative chlorine atom. The substrates of interest continue to grow and have expanded to cellulosic substrates, including wood [6,7].

In the context of proteins, multiple amino acids have been shown to react with these electrophiles by way of nucleophilic sidechains that appear on lysine, cysteine, serine, threonine, histidine, arginine, and tyrosine [8,9,10,11]. For example, Gotoh and coworkers showed that PEG-dichlorotriazine reacts with the lysine, tyrosine, and histidine residues of silk fibroin [8].

The re-emergence of interest in triazine chemistry derives, in large part, from functional proteomics. Weerapana’s group used dichlorotriazines bearing a propargyl amine to favor a reaction with lysine residues over cysteine [12]. Subsequently, click chemistry with rhodamine azide facilitated target identification.

To modulate the reactivity to specifically target specific residues, monochlorotriazines can be supplanted by less reactive species. Recently, three monochlorotriazines were evaluated and compared with a series of electrophilic tags [13]. Jiang and Li’s labs described the selective functionalization of tyrosine residues when the chlorine atom of a dialkoxymonochlorotrazine is replaced with pyridine [14]. Fort’s group employed a triazine bearing a carbohydrate, alkyne, and auxiliary amine to selectively target carbohydrate-binding proteins [15]. The displacement of the carbohydrate transfers the triazine label to the protein target.

All of these studies benefit from triazines that have sufficient solubility in aqueous solutions, i.e., either the cellular milieu or buffer. Indeed, the reactive, dichlorotriazine Procion dyes present numerous sulfate groups to counterbalance hydrophobic aromatic chromophores.

Hydrophobic constructs have not received significant attention. The incorporation of an indole on a triazine core affords an opportunity to develop methods for poorly soluble electrophiles for future protein functionalization studies. The indole also provides a useful spectroscopic probe to investigate proteins in vitro [16]. Its uses vary from simple spectrophotometric quantification of protein concentration to more elaborate techniques, including fluorescence anisotropy, which can report on protein aggregation [17], or changes in intrinsic fluorescence, which can report on the thermodynamics of weak binding interactions [18].

Nature adopts a conservative approach to the distribution of the indole-containing amino acid tryptophan; that is, it is incorporated into proteins less often than any of the other 20 naturally occurring amino acids. It appears at a frequency of ~1%, whereas a statistical distribution of 20 amino acids would result in 5% incorporation [19]. Similarly, tryptophan appears on the surface of proteins at a similar frequency of ~1%, i.e., only slightly more frequently than cysteine and methionine. When comparing the appearance of tryptophan in disordered protein regions to structured regions, its distribution is more skewed toward structured domains than any other amino acid except cysteine [20]. This observation emphasizes the need for chemistries that install indoles on amino acids like lysine, which are found in higher abundance on the surface of proteins and in functional disordered protein regions.

**Figure 1:**
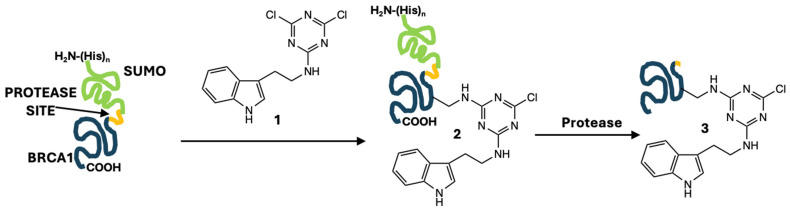
Modification of the BRCA1 domain commences with reaction with **1**, followed by peptide hydrolysis with intervening separation steps. The protein construct comprises the desired BRCA1 domain (blue), as well as an *N*-terminal SUMO tag (green) attached via a proteolytic linker (yellow). Reaction with the amine of the single BRCA1 lysine is shown.

Site-directed mutagenesis offers one strategy for the incorporation of tryptophan and its indole reporter into proteins at the genetic level. However, it may not be the most convenient option when one wishes to install an indole onto a library of variants that have already been generated through directed mutagenesis. Here, we report the incorporation of an indole onto a lysine residue. Our strategy requires only a short modification reaction and one additional buffer exchange during protein purification using a simple, inexpensive labeling agent, **1**, a dichlorotriazine with a pendant tyramine (Figure 1). The protein domain of interest (blue in Figure 1) is initially incorporated into a larger protein construct, which facilitates the purification and removal of the potentially modified *N*-terminus. leaving the domain of interest with a single indole addition.

Our specific target is the disordered PALB2-binding domain of BRCA1, which presents a single lysine sidechain [21]. Installation of an indole is potentially useful for investigating domain function as it does not have native tyrosine or tryptophan residues for measuring absorbance or fluorescence. Naturally occurring variants in this domain of BRCA1 have been associated with disruption of PALB2 binding and loss of tumor-suppression function [22,23]. Specifically, loss of the BRCA1/PALB2 interaction due to mutation of either protein leads to decreased efficiency of DNA-repair through homologous recombination, the least error-prone pathway for repair of double-stranded DNA breaks [23]. This loss of efficiency then leads to the accumulation of DNA damage over time and increases the risk of mutation of an oncogene or tumor-suppressor gene increasing cancer risk. In addition to implications in tumorigenesis, cancer cells containing variants which disrupt the BRCA1/PALB2 interaction are more susceptible to specific chemotherapeutics such as cisplatin and Olaparib, highlighting the importance of this interaction in breast cancer treatment as well [23]. Given the existing library of variants already generated to investigate the effects on binding function, the installation of a fluorophore to this domain at the protein level is desirable [24].

## 2. Materials and Methods

Synthesis of **1**: Cyanuric chloride (0.434 g, 2.35 mmol) was dissolved in 23.5 mL of tetrahydrofuran (THF) and cooled to −10 °C. Subsequently, tryptamine (0.377 g, 2.35 mmol) was dissolved in 23.5 mL of THF and added to the reaction mixture dropwise, followed by 6 mL of 1 M NaOH. The reaction mixture was allowed to warm to room temperature and stirred for 2 h. The reaction was diluted with 100 mL of water and extracted three times with 50 mL portions of ethyl acetate. Organic fractions were combined and removed in vacuo to give **1** as a white solid (0.480 g, 60% yield). ^1^H NMR and ^13^C NMR confirmed the formation of the product. ^1^H NMR (DMSO- *d_6_*, 400 MHz): δ 10.85 (s, 1H), 9.28–9.25 (t, *J* = 6 Hz, 1H), 7.60–7.58 (d, *J* = 8 Hz, 1H), 7.36–7.33 (dt, *J* = 8 Hz, *J* = 1 Hz, 1H), 7.20–7.19 (d, *J* = 2 Hz), 7.10–7.06 (dd, *J* = 9 Hz, *J* = 1 Hz, 1H), 7.01–6.97 (dd, *J* = 7 Hz, *J* = 1 Hz, 1H), 3.59–3.53 (dt, *J* = 7 Hz, *J* = 6 Hz, 2H), 2.97–2.93 (t, *J* = 8 Hz, 2H). ^13^C{^1^H}NMR (DMSO-*d_6_*, 100 MHz): δ 169.9, 168.9, 165.6, 136.7, 127.6, 123.5, 121.5, 118.8, 111.9, 111.4, 42.1, 24.7.

BRCA1 construct preparation: BRCA1 residues 1377–1426 (UniProt P38398-1) were cloned into a kanamycin-resistant bacterial expression vector on the C-terminal side of a histidine-tagged SUMO downstream of a H3C-protease recognition site. *Escherichia coli* (BL21 DE3) cells were then transformed with this BRCA1-containing plasmid, plated on LB agar plates containing 10 μg/mL of kanamycin, and incubated at 37 °C overnight. The transformed cells were grown to an optical density at 600 nm of 0.6–0.8 in LB media containing kanamycin antibiotic. Protein expression was induced with 0.2 mM IPTG at 16 °C for 16 h. The harvested cells were resuspended in 0.5 M NaCl, 20 mM TRIS pH 7.4, 5 mM imidazole, protease inhibitor cocktail (Sigma, St. Louis, MO, USA), 0.5 mg/mL lysozyme, and 0.5 mg/mL DNase and then lysed using sonication. The lysate was clarified by centrifugation and his-tagged SUMO-fusion protein was removed using a Talon Crude cobalt column on an Äkta Start (GE Healthcare, Chicago, IL, USA). Imidazole was removed via dialysis in 25 mM phosphate buffer, 50 mM NaCl, pH 6.5 at 4 °C for 16 h. PALB2 residues (UniProt Q86YC2) were similarly cloned into a bacterial expression vector downstream of an H3C cleavable His-tagged SUMO and purified identically.

BRCA1 construct modification: A solution of **1** (40 mg) is dissolved in 1 mL of DMF and added to the fusion protein in 1 mL of 120 mM sodium borate buffer at pH 9.4. These amounts correspond to 200 equivalents of **1** to protein. Instantly, a white precipitate forms, which is separated from the reaction mixture after 10 min via centrifugation for 10 min at 3000 RPM at 4 °C, with the resulting supernatant centrifuged again for 10 min at 15,000 RPM at room temperature. The supernatant is dialyzed overnight into 150 mM NaCl, 25 mM phosphate buffer pH 6.5 at 4 °C.

BRCA1-**1** cleavage and removal of SUMO from protein products: SUMO-fused proteins were cleaved using GST-tagged H3C protease for 1 h at room temperature in the presence of dithiothreitol. Cleavage was judged to be >95% complete by SDS-PAGE in all cases. BRCA1 and PALB2 constructs were purified using glutathione agarose resin to remove the GST-tagged H3C protease and HisPur Ni-NTA resin to remove histidine-tagged SUMO. Any trace protein impurities were removed using size exclusion chromatography in 50 mM NaCl, 25 mM phosphate buffer, pH 6.5.

BRCA-**1** quantification: Concentrations of BRCA1 were estimated from the peptide bond absorbance at 205 nm using a Nanodrop one^C^ (Thermo Fisher, Waltham, MA, USA) and Scopes calculation [25]. While protein backbone absorbance readings lacked accuracy and precision (typically underestimated BRCA1 concentrations), the absence of aromatic amino acids necessitates this protocol. Concentrations were corrected using densitometry from SDS-PAGE bands quantified with ImageJ when needed.

Circular dichroism spectroscopy: Circular dichroism (CD) spectra were collected on a JASCO J-810 spectrophotometer (Jasco Corp., Tokyo, Japan). Spectra were recorded at room temperature from 260 nm to 190 nm using a 1 mm quartz cell and 1 nm resolution with a scan rate of 100 nm/min. Two scans were recorded and averaged for each sample. Raw data were manipulated via the subtraction of appropriate background spectra and smoothed using manufacturer provided software. Graphs were produced with GraphPad PRISM. All samples were in 8.4 mM TRIS, 3.95 mM phosphate buffer, 7.9 mM NaCl, pH 7.0. Ellipticity (Δε) was calculated using Equation (1) [26], where θ is the raw ellipticity data (mdeg), *C* is concentration (M), *l* is the pathlength of the cell (mm), *n* is the number of amino acids, and 3298 is the corrective value. For both samples, *l* = 1, *n* = 54, and *C* = 19 μM for BRCA1 control and 12 μM for modified BRCA1.

(1) $$\Delta \varepsilon =\frac{\theta }{C\cdot l\cdot \left(n-1\right)\cdot 3298}$$

Fluorescence: Fluorescence spectra were measured with a Cary Eclipse spectrophotometer (Agilent Technologies, Santa Clara, CA, USA) with a photomultiplier tube voltage of 950 V in a 0.4 mm quartz cuvette with a resolution of 1 nm. Fluorescence measurements were carried out as follows: excitation and emission slit widths were 5 mm and 5 mm; excitation wavelength was set at 295 nm. Protein samples (10 μM) in 50 mM NaCl, 25 mM phosphate buffer, pH 6.5, were used for the measurements. After subtraction of the background spectra, the resulting spectra were smoothed using a moving average of ten points using GraphPad PRISM (version 10).

Isothermal titration calorimetry: Samples of purified PALB2 were prepared for isothermal titration calorimetry (ITC) using concentrations determined from absorbance at 280 nm and the theoretical extinction coefficient of the single tyrosine residue in this construct. BRCA1 concentrations estimated from absorbance at 205 nm (see BRCA1 quantification above) were corrected using a 1:1 stoichiometry from the fit of ITC data as well as quantification of band intensities on SDS-PAGE using densitometry with ImageJ. The 1:1 stoichiometry of BRCA1 and PALB2 is supported by a solution structure of the heterodimer in the protein data bank (PDB ID 7K3S), and the correlation time is measured by NMR via the method of Song et al. [27], both using similar-length constructs of BRCA1 and PALB2 derived from mouse.

ITC measurements were performed using a Malvern Microcal ITC_200_ (Malvern Panalytical, Malvern, UK) with a rotating syringe at 300 rpm and at 25 °C. Both protein samples were in buffer containing 50 mM NaCl and 25 mM sodium phosphate buffer system at pH 6.5. Control BRCA1 at 0.6 mM was titrated into PALB2 at 0.06 mM in the cell for a series of 17 injections. Modified BRCA1 was at lower concentration (0.255 mM), which was compensated for by performing 32 injections into PALB2 at the same concentration as the control (0.06 mM). Data were fit to a single binding site model using standard procedures described in the instrument manual with Origin software (Origin Lab, Northampton, MA, USA) to obtain thermodynamic parameters. Graph images were produced using GraphPad Prism.

Mass spectrometry: The purified, unmodified BRCA1 and the modified construct were first lyophilized and then reconstituted with 0.01% formic acid in water. A data-dependent LC-ESI-MS/MS mode of acquisition was performed on a LTQ Orbitrap Velos Pro mass spectrometer coupled to an EASY nLC-1000 systems fitted with an EASY-Spray source (Thermo Fisher Scientific, San Jose, CA, USA) [17]. Nanoflow separations were achieved with a Phenomenex bioZen column (Phenomenex, Torrance, CA, USA) with 15 cm × 75 μm i.d. and packed with 2.6 µm PeptideXB-C18 particles and attached to a 7 µm ID nanoflow EASY spray emitter (Thermo Fisher, Waltham, MA, USA). Samples were eluted at 300 nL/min flow rate with an 80 min binary solvent gradient: solvent A and solvent B were water and acetonitrile, respectively, with 0.1% (*v*/*v*) formic acid. A total of 5 µL of the reconstituted samples was injected at stable column pressure set at 450 bar for 20 min for column equilibration purposes at 100% A. Then, the proteins in the sample were eluted using the following gradient: (i) 3 min isocratic at 5% B; (ii) linear program to 40% B over 55 min; then (iii) isocratic at 40% B for 5 min; (iv) to 90% B over 5 min; (v) isocratic at 90% B for 2 min; and (vi) resetting to 5% B in 10 min. A source voltage of 2.2 kV and ion-transfer tube temperature of 275 °C were used. During elution, full-scan mass spectra (MS) were acquired with a nominal resolution of 60,000 (at *m*/*z* 400) in the Orbitrap, and up to 20 MS-dependent tandem mass spectra (MS/MS) were obtained in the ion trap. Each full MS/MS spectrum was acquired using collision-induced dissociation (CID) of only multiply charged ions (z ≥ 2). After the selection of the ion to be fragmented, dynamic exclusion was set for 60 s.

## 3. Results and Discussion

**Figure 2:**
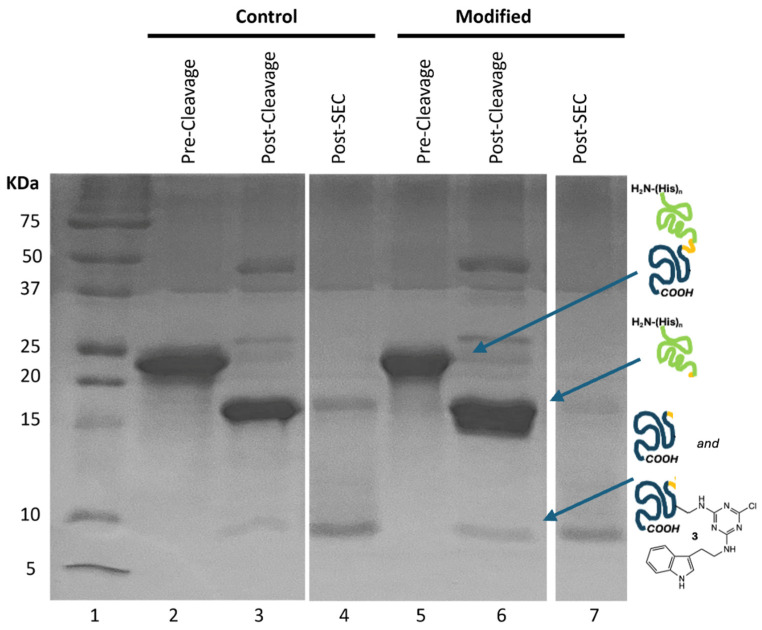
Modification of the BRCA1 domain commences with functionalization of lysine with **1**, followed by peptide hydrolysis with intervening separation steps. Lane 1 shows molecular weight markers. Lanes 2 and 5 are the isolates following purification with the SUMO-tag. Lanes 3 and 6 show the results of the cleavage reaction. Lanes 4 and 7 show the isolated BRCA1 construct after size exclusion chromatography (SEC).

The gel shown in Figure 2 traces the chemistries employed to generate the desired labeled BRCA1 domain. Briefly, we start with a fusion construct bearing the SUMO domain and a peptidase site (Figure 1). SUMO is a widely used solubility tag, and the *N-*terminal histidine tag aids in purification from bacterial proteins via metal affinity chromatography. While this particular target of interest is soluble in the absence of SUMO, the protease-cleavable SUMO domain presented an opportunity to remove the *N*-terminus that likely undergoes modification in addition to the lysine residues; thus, SUMO serves as a protecting group for the *N-*terminus of the BRCA1 domain. While use of a viral protease was effective for this target, use of SUMO-fusion also presents an opportunity for cleavage using a protease specific to the three-dimensional structure of SUMO, which could be useful for targets that are more susceptible to internal proteolysis than BRCA1. Figure 2 shows that the fusion construct is effectively purified from bacterial proteins in a single chromatography step (lanes 2 and 5).

To affect the reaction, **1** is dissolved in DMF to provide approximately a 200-to-1 mole ratio of **1** to protein. Upon addition to a buffered solution of protein at 4 °C, precipitation is observed. After 10 min, the reaction mixture is centrifuged and the pellet of residual **1** is removed. The cloudy supernatant is centrifuged a second time. The supernatant remaining is shown in lane 5. ^1^H NMR spectroscopy confirms that the precipitate is unreacted **1**. As a control, DMF without **1** was added to a protein preparation. Experiments involving this preparation are referred to as “control”.

Next, H3C protease is used to cleave between the SUMO and BRCA1 domains. SUMO presents 19 lysine residues. While these residues likely serve as sites for reaction, derivatization with **1** does not appear to preclude the ability of the protease to catalyze hydrolysis (lanes 3 and 6).

The products of hydrolysis are separated using Ni-affinity chromatography: SUMO domain has a poly(His) tag. Reports have indicated that dichlorotriazines will react with histidine residues [8]. While this modification could interfere with purification, its failure to do so suggests either selectivity for lysine or incomplete modification of the tag.

**Figure 3:**
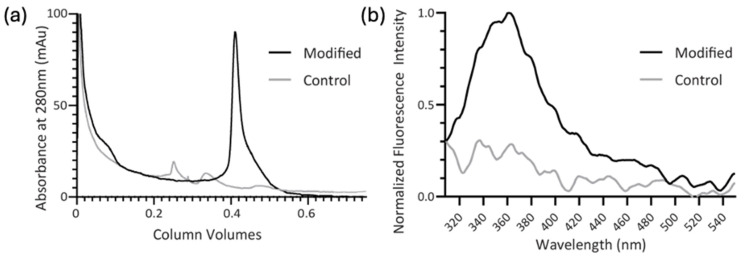
Absorbance and fluorescence indicate reaction of BRCA1 with **1**: (**a**) size exclusion chromatograms showing modified BRCA1 with absorbance at 280 nm and the control BRCA1 preparation; (**b**) the fluorescence spectra of the purified construct modified with **1** and the control BRCA1 preparation. Fluorescence intensity is normalized to the maximum of the modified sample.

Subsequently, size exclusion chromatography yields the desired constructs (lanes 4 and 7). The chromatograms provide evidence for successful reaction. Figure 3a shows that the protein eluting at the size of BRCA1 shows absorbance at 280 nm due to a lack of native aromatic residues in this construct this wavelength reports solely on the presence of the indole. By comparing the ratios of absorbance at 215 nm and 280 nm, the extent of functionalization is believed to be as high as 75%. Batch-to-batch variation is observed to be 40–75% modification.

Throughout, we note that the desired species appears to run at a higher molecular weight than predicted, as determined by SDS-PAGE. This phenomenon has been documented with natively disordered proteins and is attributed to these domains having fewer hydrophobic residues and more negatively charged residues, which inhibit the interactions of the domain with SDS [28].

Fluorescence spectroscopy of the purified BRCA1 after modification provides additional evidence of indole attachment. The characteristic indole fluorescence signal is observed only in the modified protein sample and not in the otherwise identically treated control (Figure 3b). Typical tryptophan emission (which originates from its indole moiety) varies from ~320 to 360 nm, largely based upon the environment of the residue, with 360 nm representative of free tryptophan in solution [29]. The indole emission observed here (ca. 360 nm) is consistent with the predicted disordered BRCA1 construct in the absence of the binding partner, PALB2.

**Figure 4:**
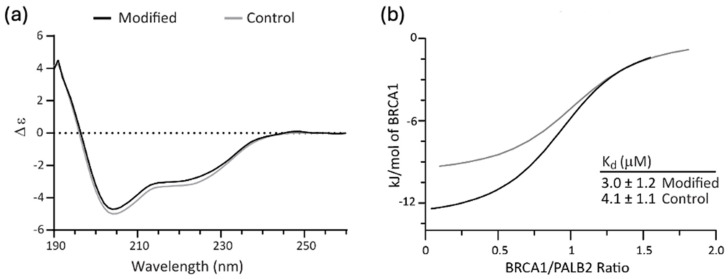
Indole addition does not significantly affect BRCA1 structure or function: (**a**) CD spectra of modified BRCA1 (black) and control BRCA1 (gray); (**b**) best fit binding isotherms describing ITC titration of modified BRCA1 (black) and control BRCA1 (gray) into PALB2. The dissociation constants derived from the fit are shown.

CD spectroscopy was used to determine whether functionalization induced structural changes to the disordered domain. Some impact on the structure of the BRCA1 domain might be expected given the hydrophobic nature of the indole and its ability to engage in π-π, π-cation and hydrogen bonding interactions. The CD spectra reported in Figure 4a revealed that modified BRCA1 domain had the same secondary structure conformation as the unmodified protein. Based on typical peptide behavior, both spectra are indicative of random coil with some helical propensity [30].

ITC was used to determine whether functionalization of BRCA1 affected its binding to PALB2 (Figure 4b and Figure S1). Upon binding PALB2, the disordered domain takes on a helical structure to mediate hydrophobic interactions between partners by adopting a leucine zipper motif (PDBID 7K3S) [31]. Interference with binding could result from steric effects, disruption of the desired hydrophobic interactions, or removal of the cationic lysine sidechain that engages in ion pairing across the interface. We find that while more heat is released upon binding of the modified BRCA1 to PALB2 (see Table S1 for fit parameters), the binding constant of the modified BRCA1 is not significantly different from that of the unmodified construct, with K_d_ values of 3.0 μM and 4.1 μM, respectively. The change in heat without a corresponding difference in binding affinity indicates enthalpy-entropy compensation in the modified protein, which is a noted feature of intrinsically disordered protein–protein interactions [32]. The similarity in binding affinity indicates that while native tryptophan is notorious for mediating protein–protein interactions (both in terms of statistical presence in binding interfaces and contribution to energetics), it may not drive binding in the absence of a co-evolved binding interface on the heterodimeric partner.

**Figure 5:**
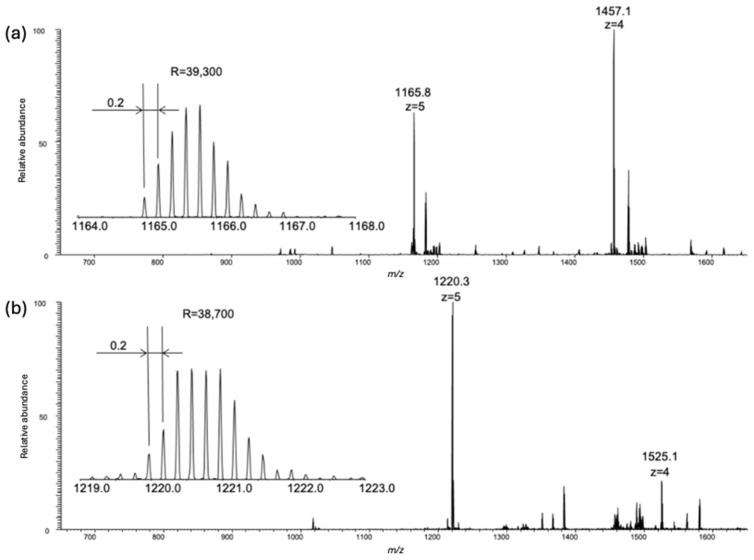
Electrospray ionization (ESI) mass spectra of the (**a**) unmodified and (**b**) modified BRCA1 construct. Insets show that *m*/*z* 1165.8 and *m*/*z* 1220.3 are quintuple-charged ions (z = 5, with R indicating mass resolution), which confirm the modification of GPGSSVSEDC SGLSSQSDIL TTQQRDTMQH NLIKLQQEMA ELEAVLEQHG SQPS (**a**) with the 272 Da indole incorporation (**b**).

Nanoflow LC-ESI-MS and MS/MS were employed to verify the reaction, quantify the number of additions, and identify the site of modification (Figures S3–S5) [33]. The recorded ESI-MS spectra revealed that the expected modification is observed when comparing the unmodified control (Figure 5a) with the reaction product (Figure 5b).

Unfortunately, MS/MS spectra (Figure S4) were not informative in narrowing the site of modification. This sequence (shown below) corresponds to residues 1377–1426 of the BRCA1 and contains four additional amino acids on the *N*-terminus (underscored GPGS) that result from expression and enzymatic cleavage. It contains a single lysine residue (underscored K).

GPGSSVSEDC-SGLSSQSDIL-TTQQRDTMQH-NLIKLQQEMA-ELEAVLEQHG-SQPS

Modification of lysine 44 in the sequence (corresponding to residue 1406 in BRCA1) could not be uniquely identified due to the fragment-directing effect of the proline residue at position 53 (underscored penultimate P at the C-terminus) [34]. Gratifyingly, the mass spectra of the 52 amino acid sequences of the control and reaction product also differed by 272 Daltons, corresponding to the incorporation of a single indole. Evidence for two substitutions arise from searching the chromatogram for the expected ion, which can only be detected at 1% abundance compared with the singly substituted product (Figure S5), leading us to conclude that modification of the histidine residue or seven serine residues is unlikely.

## 4. Conclusions

Despite possessing no solubility in water, descriptively, dichlorotriazine **1** can be reacted with the disordered domain of BRCA1 to install a fluorescent handle. The high reactivity of **1** and presumed global reaction with the protein construct did not adversely affect the ability of the protease to perform cleavage nor the poly(His) tag to facilitate separation. Modification of the PALB2-binding domain of BRCA1 did not significantly affect its structure or function, allowing for the use of modified BRCA1 as a fluorescent probe. This methodology forms the basis for generating materials for the purposes of proteomics or the development of high-throughput assays for screening naturally occurring variants that may result in the disruption of the BRCA1/ PALB2 interface. The reactivity of **1**, however, leads to a noteworthy limitation: this strategy is intended for targets with a single reactive site or targets where multiple modifications are desirable. The incorporation of multiple labels would be expected for targets containing multiple reactive sites.

## Data Availability

The data presented in this study are available within the manuscript and the supporting material. Any additional data are available upon request.

## Supplementary Materials

The following supporting information can be downloaded at: https://www.mdpi.com/article/10.3390/biom14121625/s1: General methods and calculations: Figure S1. Isothermal Calorimetry Experiments; Figure S2. SDS Gel showing protein purification; Figure S3. The LC chromatograms; Figure S4. The LC–ESI-MS total-ion current (TIC) chromatograms; Figure S5. MS/MS does not reveal the site of modification; Figure S6. Evidence for construct that underwent two reactions with 1; Table S1. Parameters derived from ITC analysis thermodynamic binding parameters derived from fitting ITC data.
