# Supplementary material for: Installation of an Indole on the BRCA1 Disordered Domain Using Triazine Chemistry
Source: Biomolecules. 2024 Dec 18;14(12):1625. doi: 10.3390/biom14121625 (PMC11726873; doi:10.3390/biom14121625)

SUPPORTING INFORMATION

Installation of an indole on the BRCA1 disordered domain using triazine chemistry

Liam E. Claton [1,†], Chrissy Baker,[2,†] Hayes Martin,[2] Sergei Dzyuba,[1] Khadiza Zaman,[3] Laszlo Prokai,[3] Mikaela Stewart,[2] and Eric E. Simanek[1,]*

Table of Contents

Isothermal Calorimetry

Method for Isothermal Calorimetry S2
Figure S1. Isothermal Calorimetry Experiments S3
Table S1. Parameters derived from ITC Analysis S3

Protein Chemistry

Protein Expression in *E. coli* S4
BRCA1-SUMO Purification S5
Protein Modification S7
Proteolysis S7
Nickel Affinity Chromatography S7
Gel Electrophoresis S8
Figure S2. SDS Gel Showing protein purification S9
Gel Filtration Chromatography S10
Figure S3. LC Chromatograms S10

Mass spectrometry

Discussion S11
Figure S4. The LC chromatogram S11
Figure S5. MS/MS does not reveal the site of modification S12
Search for Multiple Substitutions S13
Figure S6. Evidence for construct that underwent two reactions with **1** S13

Method for Isothermal Calorimetry

Samples of purified PALB2 were generated for ITC using concentrations derived from absorbance at 280 nm and the theoretical extinction coefficient of the single tyrosine residue in this construct. Concentration of BRCA1 protein samples for ITC were estimated from the backbone absorbance at 205 nm. While we found that backbone absorbance readings lacked accuracy and precision (typically underestimated BRCA1 concentrations), the lack of aromatic amino acids necessitates this protocol. BRCA1 concentrations were then corrected using a 1:1 stoichiometry from the fit of ITC data as well as quantification of band intensities on SDS-PAGE using ImageJ. The 1:1 stoichiometry of BRCA1 and PALB2 is supported by a solution structure of the heterodimer in the protein data bank (PDB ID 7K3S) and correlation time measured by NMR (Song et al 2018) both using similar length constructs of BRCA1 and PALB2 derived from mouse.

ITC measurements were performed using a Malvern Microcal ITC_{200} with a rotating syringe at 300 rpm and at 25°C. Both protein samples were in buffer containing 50 mM NaCl and 25mM sodium phosphate buffer system at pH 6.5. Control BRCA1 at 0.6 mM was titrated into PALB2 at 0.06 mM in the cell for a series of 17 injections. Modified BRCA1 was at lower concentration (0.255 mM) than the control which was compensated for by performing 32 injections into PALB2 at 0.06 mM. Data were fit to a single binding site model using standard procedures described in the instrument manual with Origin software to obtain the thermodynamic parameters of binding shown in Table S1. Graph images were produced using GraphPad Prism.

Figure S1. Isothermal Calorimetry Experiments. Isothermal Titration Calorimetry experiments measuring the titration of BRCA1 constructs into the BRCA1-binding domain of PALB2. Panels **A & B** resulted from titration of BRCA1 control (0.6 mM) into PALB2 (0.06 mM). Panels **C & D** resulted from titration of BRCA1 Modified (0.3 mM) into PALB2 (0.06 mM). Panels **A & C** provide the heat exchange for each individual injection. Panels **B & D** show enthalpy change of each injection versus molar ratio of BRCA1 to PALB2. Solid curves show the fit of data to a single site model of the binding isotherms.

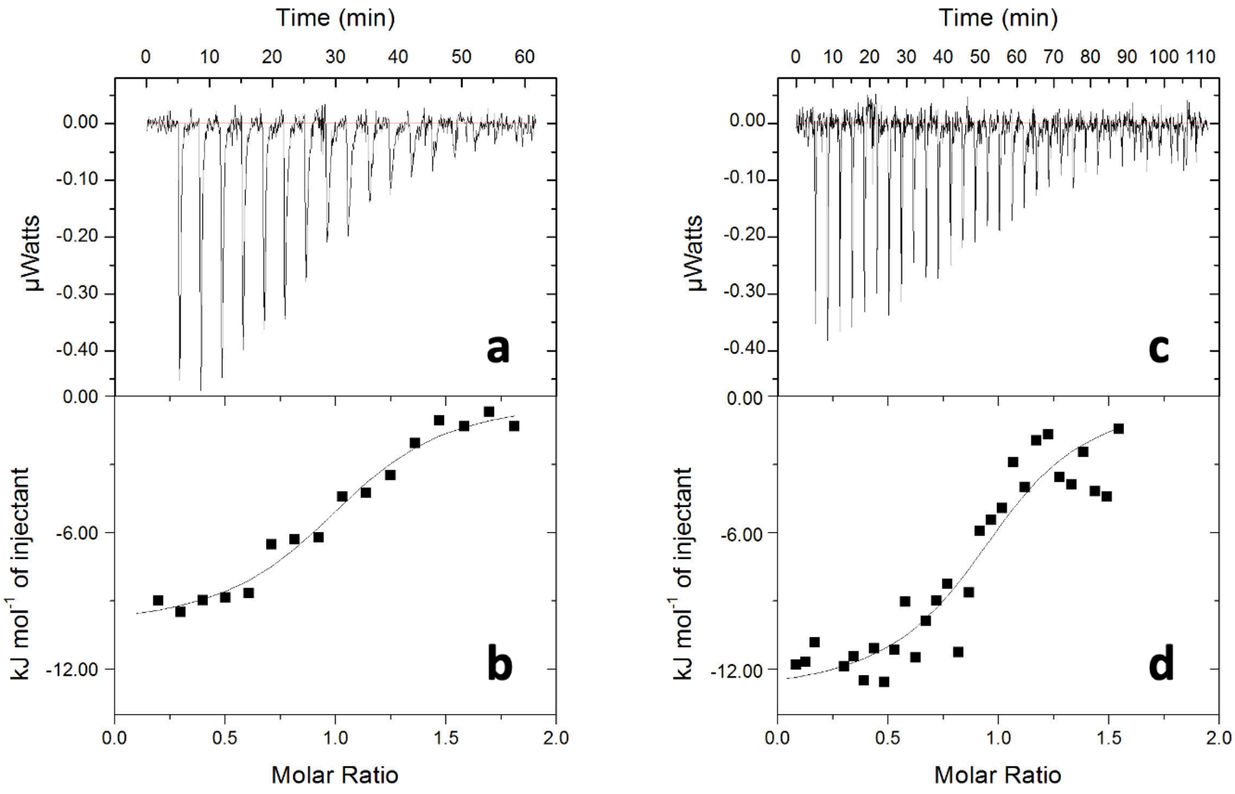

Table S1. Parameters derived from ITC Analysis

Parameters derived from fit	Control	Modified
N	1.02 ± 0.03 Site	1.01 ± 0.04 Site
K_a	$2.5 \times 10^5 \pm 7 \times 10^4$ M^{-1}	$3.4 \times 10^5 \pm 1.4 \times 10^5$ M^{-1}
ΔH (significant difference)	-1.03×10^4 ± 500 J/mol	-1.31×10^4 ± 800 J/mol
ΔS	68.8 J/mol/deg	62.0 J/mol/deg

Protein Expression in *E. coli*

DAY 1

Roughly 20 μL of a frozen stock of BL21 (DE3) *E. coli* cells in 35% glycerol previously transformed with the described BRCA1 or PALB2 construct was added to 300 μL of sterile LB broth. This solution was spread onto an LB agar plate containing kanamycin using sterile beads. The bacteria were left to grow overnight at 37°C.

DAY 2

Sterile LB broth (4 L) was prepared by the following procedure: 25 g of LB nutrient powder and 1 L of distilled water were each added into four baffled 2.8 L Erlenmeyer flasks. The LB broth in the flasks were autoclaved for 30 minutes.

Sterile LB broth (3 mL) was added to the agar plate and a sterilized spreader was used to scrape and suspend the colonies in the broth. This suspension was transferred into a 15 mL conical tube and then equally distributed into the four 2.8 L Erlenmeyer flasks of LB broth. Flasks were shaken at 37 °C at 250 RPM for roughly two hours until optical density reached 0.5-0.6. Cultures were cooled to 16°C for one hour and then 200 μL of 1M IPTG stock was added to each flask for 0.2 mM final IPTG concentration. Protein expression was then induced for 15-16 hours overnight at 16°C.

DAY 3

Cells were harvested by centrifugation at 3500 RPM and the cells from 2 L were resuspended together in 25 mL of metal affinity buffer A according to the TALON Crude 5 mL column protocol. The cell suspensions were stored at -80 °C until purification.

BRCA1-SUMO Purification

DAY 1

The cell suspension from 2 L of over-expression was thawed in a bath of room temperature water and treated with 300 µL of 100X Protease Inhibitor Cocktail (PIC), ~5 mg of lysozyme, and ~5 mg of DNAse. The solution was transferred into a glass 50 mL beaker and sonicated for 30 total minutes with intervals of 15 seconds of pulse and 30 seconds of rest (10 minutes of total pulse time). The now lysed cells were transferred into a 40 mL centrifuge tube and centrifuged at 14000 RCF at 4°C for 25 minutes.

The ÄKTA Start GE chromatography system used has a sample line and two buffer lines. First, the sample line was cleaned with degassed water. Both buffer lines (A and B) were placed into degassed water and line B was set to run through the system at 3 mL/min for 5 minutes. Line B was then placed into buffer B (50 mM sodium phosphate, 300 mM NaCl, 1 M imidazole, pH 7.4). %B was set to 100% and flowed at 3 mL/min for 5 minutes. %B was set to 0% and degassed water was run through line A at 3 mL/min for two minutes. With a flowrate of 1 mL/min, the TALON Crude cobalt column was attached drop to drop from the top first then the bottom. 5 column volumes (CV) of degassed water was run through the column through line A. The system was paused and line A was placed into buffer A (50 mM sodium phosphate, 300 mM NaCl, 5 mM imidazole, pH 7.4) and allowed to run for 5 CV. The supernatant lysate was transferred into a 50 mL conical tube and the sample line was placed in it. An automated method loaded the cell lysate through the column, washed with 20 column volumes of Buffer A, and then eluted the bound protein into the fraction collector using a gradient to 50% Buffer B over 15 column volumes. SDS page gel was run to confirm the location of the protein in the fractions.

Dialysis buffer preparation: DI H_2O (3.5 L in a 4 L beaker) is continuously stirred with a magnetic stir bar as 4.48 g of monobasic $NaPO_4$ monohydrate, 9.64 g of dibasic $NaPO_4$ anhydrous, and 35 g of NaCl are added to the beaker and allowed to dissolve. The pH was measured via pH meter to be 7 and the volume of the beaker is brought to 4 L using DI H_2O.

Dialysis procedure: 2 feet of Saran wrap was placed on the bench and 4 inches of 3500 Da cutoff dialysis tubing was prepared by cutting the required length away from the stock with a pair of scissors. The tubing was submerged in the dialysis buffer to hydrate it. The edge of the tube was folded over and clipped with a dialysis clip to make a sealed tube on one end. The protein solution was poured into the tube. The air was pressed out of the top opening of the dialysis tubing and then the opening was folded over onto itself and clipped to form a seal with a dialysis clip. One of the dialysis clips was fastened to a piece of foam to keep the dialysis bag from sinking to the bottom of

the beaker. The beaker with the dialysis tubing submerged was covered with the Saran wrap and placed in the 4 ℃ cold room on a stir plate set to 60 revolutions per minute and left overnight.

DAY 2

The next day, the plastic wrap was removed from the top of the beaker and laid on the bench. The dialysis tubing was removed from the beaker and one side of the tubing was unclipped while holding the tubing together to ensure no protein spilled out. The tube was then poured into a 15 mL conical tube. The protein was then concentrated to 2 mL using at 3500 cut off centrifugal concentrator. The concentrated protein was split into two microcentrifuge tubes and frozen at -80 °C to use for modification or the mock-modification protocol.

Protein Modification

A 5 mL RBF containing a magnetic stir bar and 1 mL of sodium borate buffer (0.12 M, pH 9.8) was placed in an ice bath (4 ℃). To this, 1 mL of BRCA1 with a SUMO tag (0.64 mM; in 0.025 M sodium phosphate buffer, pH 7) is slowly added via pipette. The resulting clear solution was pH 9.4 as determined with a pH meter.

Stirring commenced with the stir plate set to 60 revolutions per minute. In a 3 mL vial, 40 mg of **1** was dissolved in 1 mL of DMF and added to the reaction dropwise via pipette over 2 minutes. After the first drop, a white milky solid appeared in the solution. After 15 minutes, the entire solution took on a milky, cloudy appearance. The reaction was allowed to stir at 4 ℃ for 2 hours. After 2 hours, the solution was added to a 15 mL conical tube and centrifuged for 10 min at 3000 RPM at 4 ℃. A small white pellet formed at the bottom of the conical tube. The supernatant appeared opaque, likely still having white solid suspended. The supernatant was divided into 3 Eppendorf tubes and centrifuged at RT for 10 min at 15000 RPM. Small white pellets formed at the bottom of each Eppendorf tube. The supernatants were combined and an aliquot was subjected to TLC using 19:1 DCM:MeOH as the eluent which confirmed (upon UV irradiation) the presence of **1** in the solution. The combined supernatant was dialyzed overnight in 4L 0.025 sodium phosphate buffer pH 0.7 at 4℃ as described in BRCA1-SUMO purification above. A gel electrophoresis sample (10 μL) was taken prior to proteolysis (labeled as pre-cleavage on the gel forthcoming).

The pellet from the 15 mL conical tube was resuspended in 5 mL of EtOH and re-centrifuged at 3000 RPM for 30 min. The pellet was then placed on a watch glass and allowed to evaporate to yield 19 mg of a white crystalline solid which was confirmed to be **1** using NMR spectoscopy.

Proteolysis

To proteolyze the BRCA-SUMO fusion construct, H_3C protease was added to the modified or mock-treated protein along with 1 mM DTT in a 15 mL conical tube. The conical tube was placed on a narrator for 1 hour at room temperature. After 1 hour a gel electrophoresis sample was taken (labeled as post cleavage on the gel below).

Nickel Affinity Chromatography

HisPur Ni-NTA and GST columns were removed from the 4 ℃ fridge and placed on a clamp stand with the GST column directly over eluting into the Ni column. Both columns were washed with 3 column volumes (CV) of ddH_2O with the flow through

collected in a waste beaker. Both columns were then washed with 3 CV of buffer A with the flow through collected in a waste beaker. The protein is then loaded into the GST column that drips into the Ni column and the flow through is collected in a 50 mL conical tube. Both columns were then washed with 10 mL of buffer A and the flow through was collected in the same 50 mL conical tube. A gel electrophoresis sample was taken from the 50 mL conical tube (labeled as flow through in the gel below) and the tube was placed on ice. The Ni column was then washed with 15 ml of 30 mM imidazole in buffer A and collected in a 15 mL conical tube. A gel electrophoresis sample of the imidazole flow through was taken (labeled as imidazole), and the tube was placed on ice. The Ni column is then washed with buffer B and collected in a new 15 mL conical tube and a gel electrophoresis sample of the buffer B flow through was taken (labeled as buffer B flow through). Both columns were then cleaned with ddH_2O with flow through collected in a waste beaker before storing them in EtOH and returning them to 4 ℃.

Gel Electrophoresis

Mini-PROTEAN® TGX™ Precast 4-15% polyacrylamide gels from BioRad were ran at 195 volts for 45 minutes. Dual Xtra molecular weight ladder was loaded in one lane to approximate protein size. The gel was stained in an empty pipette box filled with a ~ 1 cm of Coomassie blue stain. The box was then microwaved for 15 seconds and put on the orbital shaker for 10 minutes. Destain (water, methanol, acetic acid) was added to cover the gel and the box was microwaved for 15 seconds and then returned to the orbital shaker. After 1 day in the destain the gel was placed on the light machine and a photo was taken.

Figure S2. SDS Gel Showing protein purification.

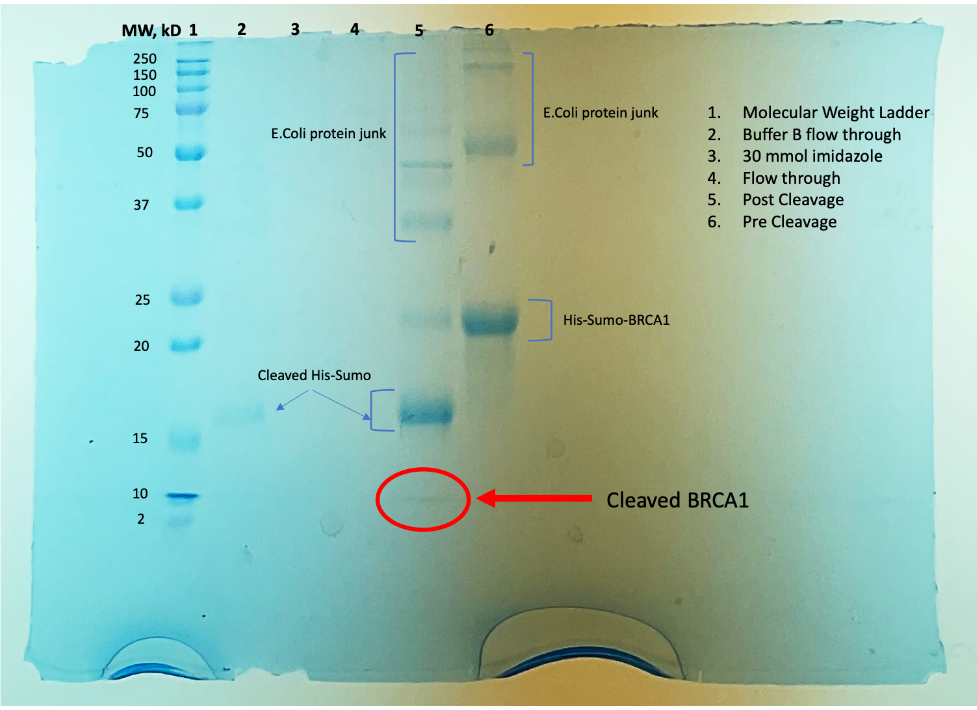

Gel Filtration Chromatography

A 20 mL sized 3k MW centrifugal concentrator was washed with ddH_2O by placing it in the centrifuge at 4°C for 5 min at 3000 rpm. The contents from the 50 mL conical tube flow through plus A wash were then added to the 3k concentrator and loaded into the centrifuge using 20 min intervals at 3000 rpm at 4°C until the volume was concentrated to ~ 2 mL. Between 20 min intervals the concentrator was gently shaken to distribute the concentrated protein through the remaining liquid.

For gel filtration, the column was first equilibrated in 50 mM NaCl, 25 mM phosphate buffer pH 6.5 by washing with 120 mL at 1 mL/min) and the sample loop was washed with 10 mL of water. 2 mL of concentrated protein from the centrifugal concentrator were then added to the sample loop. The same buffer was used for elusion at 4°C at 0.5 mL/min.

Chromatograms (shown below) identify fractions 31-36 from the gel filtration column were selected based on their UV absorbance at both 215 nm (peptide backbone) and 280 nm (indole) and concentrated down using a 6 mL 3k MW centrifugal concentrator. The concentrator was first washed with 5 mL DI H_2O by placing it in the centrifuge at 4 °C for 5 min at 3000 rpm. The water was then removed and fractions 31-33 were then added to the concentrator and spun down for 20 min at 3000 rpm at 4°C. Fractions 34-36 were then added and the concentrator was spun down in 20 min intervals at 3000 rpm until there was ~ 1 mL of concentrated protein which was added to an microcentrifuge tube and frozen at -80 °C.

Figure S3. LC chromatograms. Reaction mixture of modified protein (top), unmodified protein (bottom) monitored at 280 nm (green) and 215 nm (blue).

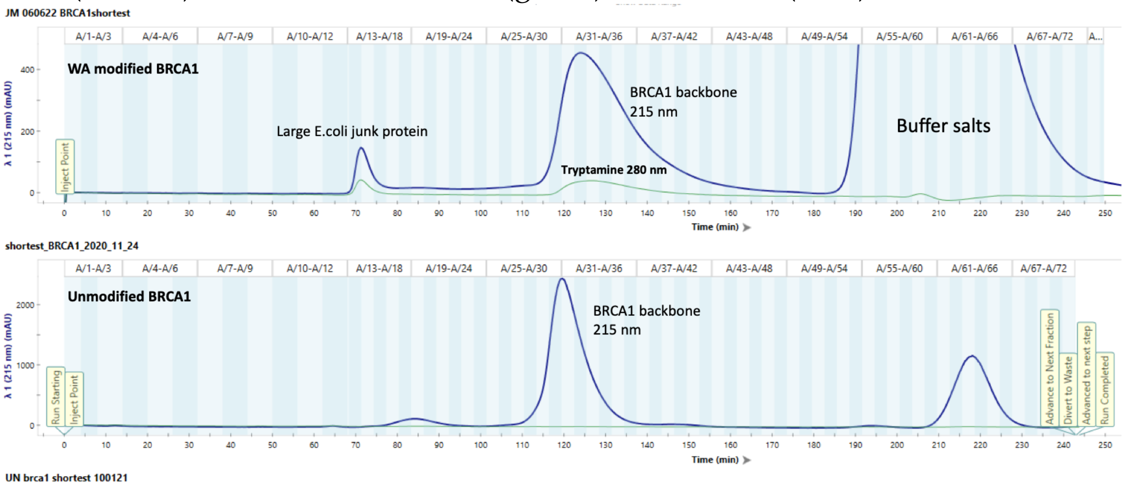

Mass Spectrometry

Data-dependent nanoflow LC–ESI-MS/MS analysis was performed on the protein samples (Figure S4).

Figure S4. The LC–ESI-MS total-ion current (TIC) chromatogram. MS/MS was performed on the 5+ (z=5) ions of the LC-separated unmodified and modified proteins (*m*/*z* 1165.8 and *m*/*z* 1220.3, respectively), but unfortunately, proline 53 of the sequence exhibited a strong fragmentation-directing effect to yield the b_{52}^{4+} ions that other sequence ions were absent. Nevertheless, the same fragmentation pattern indicates that the protein sequence was identical, with the exception being a +272-Da modification in a side chain within the sequence.

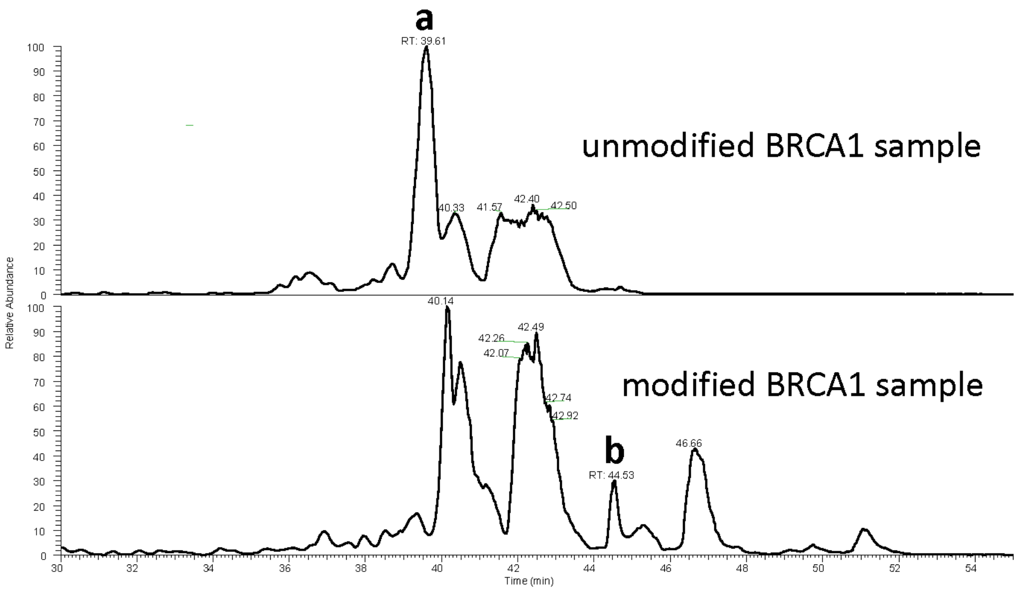

Figure S5. MS/MS does not reveal the site of modification. Top: unmodified control sequence. Bottom: Modified sequence. CID: collision-induced dissociation; 4+ and 5+ indicate positive ions with z=4 and z=5, respectively; b_{51} and b_{52} denote sequence ions according to the Roepstorff–Fohlman nomenclature (Roepstorff, P.; Fohlman, J. Proposal for a common nomenclature for sequence ions in mass spectra of peptides. *Biomed. Mass Spectrom.* **1984**, *11*, 601. doi: 10.1002/bms.1200111109).

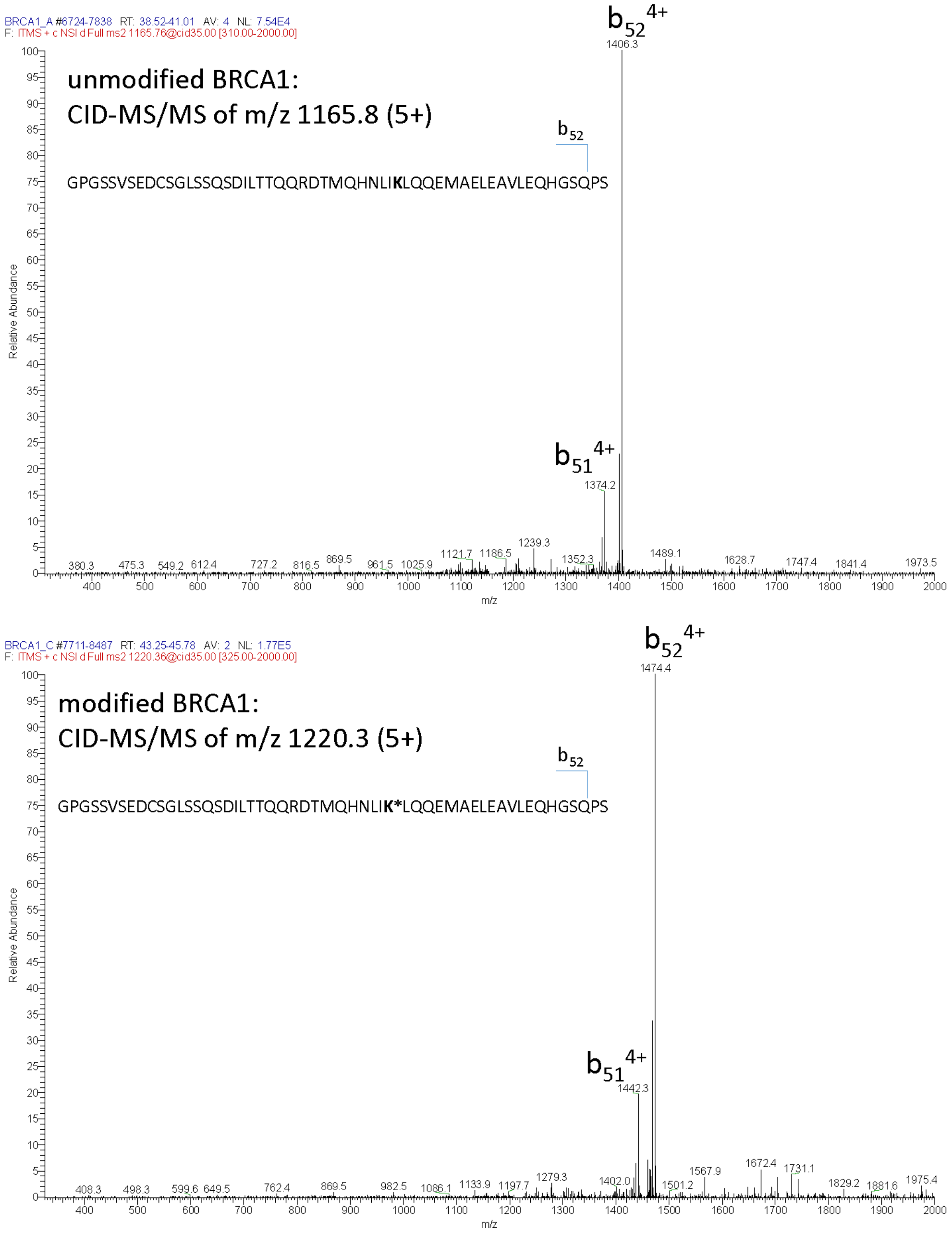

Search for multiple substitutions

A search for doubly modified construct was undertaken. This species was found but was present at about 1% of that of the singly modified protein's abundance. This can be seen in Figure S6 from the 100× magnification based on the normalization level (NL) of $2 \cdot 10^5$ in the third trace to scale the abundance of the doubly modified protein (**c**, *m/z* 1274.4 with z=5) versus NL of $2 \cdot 10^7$ for the second trace representing the singly modified protein (**b**, *m/z* 1220.3 with z=5). The low abundance of the doubly modified protein is also evidenced by the 20× magnification needed to show its molecular ion at *m/z* 1274.4 (with z=5) in the ESI mass spectrum recorded at retention time (RT) of 47.28 min.

Figure S6. Evidence for construct that underwent two reactions with **1**.

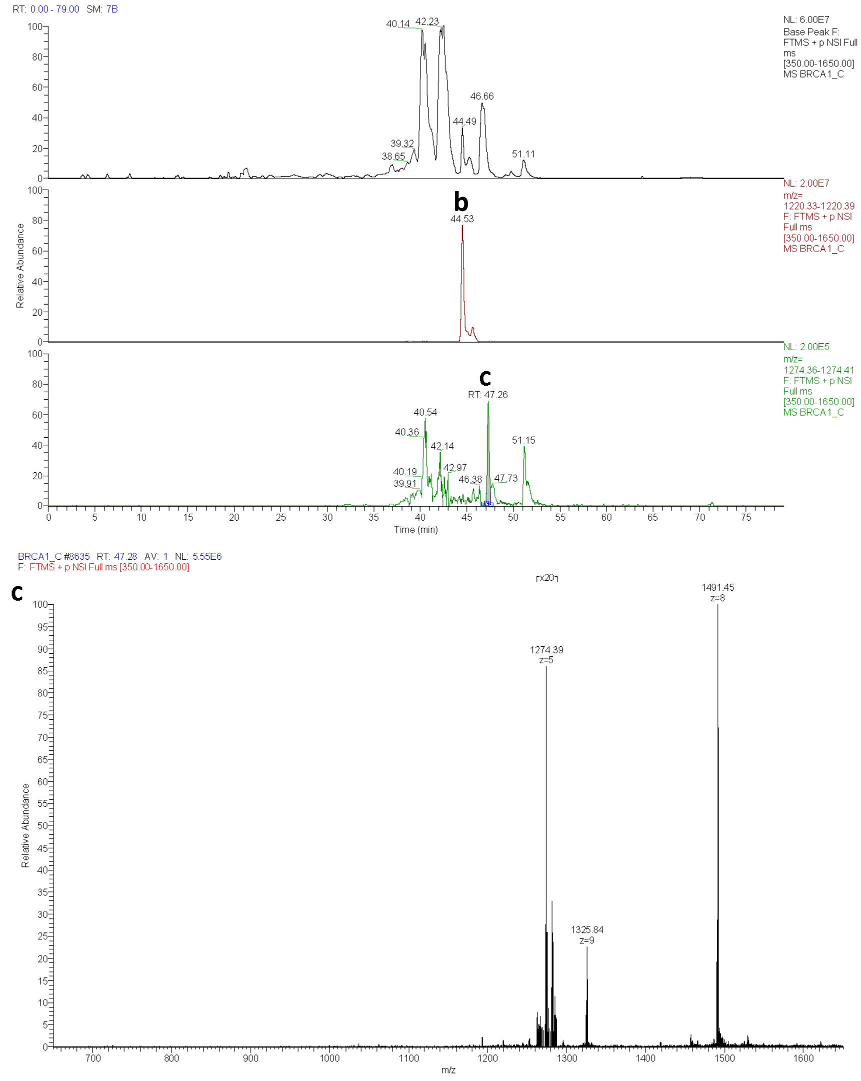

Supplement: Supplementary file 1 [file biomolecules-14-01625-s001.zip › biomolecules-3325215-supplementary.pdf]
